# Optical measurements of the twist constant and angle in nematic liquid crystal cells

**DOI:** 10.1038/s41598-024-68812-x

**Published:** 2024-07-31

**Authors:** Denitsa Bankova, Nina Podoliak, Malgosia Kaczmarek, Giampaolo D’Alessandro

**Affiliations:** 1 School of Physics and Astronomy, University of Southampton, Southampton, SO17 1BJ UK; 2 School of Mathematical Sciences, University of Southampton, Southampton, SO17 1BJ UK

**Keywords:** Mathematics and computing, Optics and photonics, Physics

## Abstract

We present a reliable optical method for measuring the twist elastic constant $\text {K}_2$ and for assessing the total twist angle in a standard nematic twist cell. The method relies on the use of a non-standard configuration of crossed polarisers and a twist cell, which allows us to measure accurately the twist-cell parameters by reducing the degeneracy between them. Grid patching and an efficient beam propagation method are utilised in the numerical models used for fitting the experimental data. The modelling shows that the polarisation dynamics in a twist cell is non-trivial and much more complex than in a planar cell. The twist elastic constant of three commonly used liquid crystals (5CB, 6CHBT and E7) was successfully extracted from cross-polarised intensity measurements.

## Introduction

Liquid crystals are widely used in display technologies and have many applications in a variety of areas including augmented reality and virtual reality^1,2^, smart windows^3,4^ and various photonic components^5^. Their endless possibilities are enabled by the fact that the liquid crystal alignment can be changed by applying an electric (or magnetic) field. The ensuing dynamic response depends on a number of parameters of the liquid crystals and of the alignment layers of the liquid crystal cells. Key examples of the former are the elastic constants: splay $\text {K}_1$, twist $\text {K}_2$ and bend $\text {K}_3$. Key cell properties are the pretilt and anchoring energy at the surface and, in the case of twist cells, the total twist. It is therefore essential for device fabrication to know accurately as many of these values as possible. Measuring the twist elastic constant $\text {K}_2$ and the total twist is particularly important for liquid-crystal displays as they are normally in a twist-cell configuration. However, measuring $\text {K}_2$ is not as easy and accurate as measuring the splay $\text {K}_1$ and bend $\text {K}_3$ elastic constants^6–8^. We have already developed a cross-polarised intensity (CPI) method for measuring the splay and bend elastic constants, viscosities, cell thickness, pretilt and polar anchoring energy^9–11^ in planar liquid crystal cells. Here, we build on this technique and extend it to twist liquid crystal cells to measure the twist elastic constant and assess the twist angle.

Many methods for measuring $\text {K}_2$ rely on determining the Fréedericksz threshold voltage or magnetic field (the critical threshold at which the liquid crystal molecules begin to reorient when an external field is applied) either optically^7,12^ or capacitively^13,14^. The twist elastic constant can be determined from the threshold field of a twist cell if the splay elastic constant ${\text {K}_1}$ and the bend elastic constant ${\text {K}_3}$ are known. Alternatively, the threshold field of a planar cell can be used to calculate $\text {K}_2$ if the field is applied along the substrates of the cell; however, a high magnetic field may be needed^7^. These methods use standard liquid crystal cells, but require a good estimate of the cell thickness and a separate measurement of the pretilt unless it is assumed to be zero. Other methods make use of liquid crystal cells with non-standard geometry, for example, a wedge cell filled with a chiral nematic^15^, a cylindrical cell^16^, a pi-cell^17^ and cells with in-plane electrodes^8,18,19^.

None of these methods are able to estimate the twist elastic constant and the twist angle of a twist cell at the same time. Therefore, a separate measurement is needed for the twist angle, which is usually determined optically^20–23^. Akahane et al.^21^ measure the twist angle and thickness of a twist liquid crystal cell placed between polarisers by minimising the intensity of the light transmitted through the system. Kuo et al.^23^ also study a twist liquid crystal cell placed between polarisers but they use a combination of transmission and reflection methods. These methods have simple setups but require the rotation of the twist cell and the analyser during the experiment, and assume a zero pretilt.

Here, a new method for determining the value of the twist elastic constant and estimating the twist angle, that has none of the listed problems, is presented. It is a simple and quick rotation-free optical method based on fitting CPI measurements. This method gives accurate results for the twist elastic constant $\text {K}_2$, the cell thickness *d* and the pretilt $\theta _0$ of a standard twist liquid crystal cell, which promotes the characterisation of transmissive liquid crystal devices in a setting very close to their desired design. In liquid-crystal displays the liquid crystal is placed between parallel polarisers in such a way that the polarisation of the incident light is parallel to the liquid crystal director (which indicates the preferred orientation of the liquid crystal molecules), but this configuration is not suitable for measuring $\text {K}_2$ as we show in our discussion. Instead, we use crossed polarisers at 45$^{\circ }$ to the liquid crystal director at the cell boundary in our experiments. This allows us to measure accurately the twist elastic constant and to limit the effect of the cell thickness and the pretilt on the measurement by reducing the degeneracy of $\text {K}_2$ and the thickness, as well as the degeneracy of $\text {K}_2$ and the pretilt. Moreover, optimising the fits allows us also to estimate the twist angle provided that it is close to 90$^{\circ }$, the standard operating regime for a twist cell.

The numerical modelling of this setup presents two computational challenges: first, how to represent uniquely the orientation of the liquid crystal as the director field in spherical coordinates is degenerate for a polar angle of 0 and $\pi$, and second, how to model the non-trivial light propagation in this system as a function of the applied voltage. We use two non-coaxial spherical coordinate systems based on previous work using grid patching^24–26^ and an effective beam propagation method based on Berreman’s formalism^27^.

The organisation of this paper is as follows. First, we present the mathematical model for a twist cell and light propagation through the cell, followed by an investigation of a suitable configuration of the experimental setup. With the appropriately chosen configuration we measure the twist elastic constant along with the splay and bend elastic constants of three commonly used liquid crystals: 5CB, 6CHBT and E7. Three important aspects of the method are discussed next: the shape of the voltage-dependent CPI of a twist liquid crystal cell, the dependence of the CPI curve on misalignment in the setup and the effect of a twist angle different from 90$^{\circ }$ on the shape of the CPI curve. The results from the new experimental method are reviewed in the conclusion.

## Mathematical model

**Figure 1:**
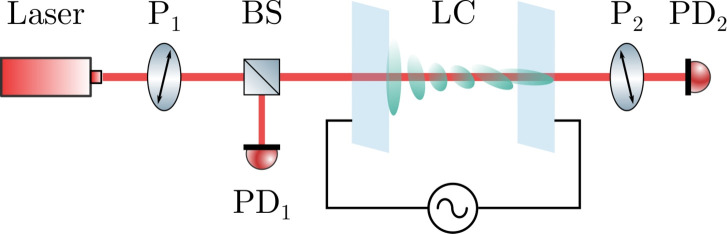
Experimental setup. The laser beam passes through the first polariser ${\text {P}_1}$, the twist liquid crystal cell (LC) and the analyser ${\text {P}_2}$. The beam splitter (BS) is used to produce reference and transmitted beams detected by the reference photodetector ${\text {PD}_1}$ and the main photodetector ${\text {PD}_2}$ respectively.

We model the experimental setup presented in Fig. 1, where a twist liquid crystal cell is sandwiched between two polarisers and the intensity of the transmitted light is measured as a function of the amplitude of the voltage applied to the cell. In general, when twist liquid crystal cells are analysed optically using crossed (or parallel) polarisers, a configuration where the polarisers are parallel or perpendicular to the liquid crystal director at the cell boundaries is used^4,28,29^. Instead, here we consider a configuration with arbitrary orientation of the polarisers with respect to the twist cell. This will allow us to explore the optimal configuration for the measurement of the twist elastic constant.

As we only need to study the bulk properties of a uniaxial nematic without flow in equilibrium conditions, we can use the Frank-Oseen static theory of nematics to model the alignment of the twist liquid crystal cell in the system in Fig. 1. The bulk (elastic and electric) free energy density for a nematic liquid crystal is^30^

1 $$\begin{aligned} {\mathscr {F}}_B=\frac{1}{2}\text {K}_1(\nabla \cdot \varvec{n})^2 + \frac{1}{2}\text {K}_2(\varvec{n} \cdot \varvec{\nabla } \times \varvec{n})^2+\frac{1}{2}\text {K}_3(\varvec{n} \times \varvec{\nabla } \times \varvec{n})^2 -\frac{1}{2}\varepsilon _0 \Delta \varepsilon (\varvec{n}\cdot \varvec{E})^2, \end{aligned}$$

where $\Delta \varepsilon$ is the difference between the dielectric coefficients $\varepsilon _\parallel$ and $\varepsilon _\perp$, $\varepsilon _0$ is the permittivity of free space, $\varvec{n}$ is the director and the applied electric field is given by $\varvec{E}$. The lateral dimensions of the cell are considerably larger than its thickness so that we can model the liquid crystal alignment as function only of the coordinate along the cell thickness, *z*. The alignment of the liquid crystal molecules in the twist cell is described by a director field, i.e. a field of unit vectors ${\varvec{n}}$ with the added property that ${\varvec{n}} \equiv -{\varvec{n}}$. It is common to use spherical coordinates to represent the unit vectors in order to preserve their norm after integration. This is the approach we take here as well, but we introduce two non-coaxial spherical coordinate systems (see Supplementary Fig. S1 online) in order to avoid coordinate singularities. The use of multiple spherical coordinate systems is based on previous work using grid patching^24–26^. The alignment equations, given as Supplementary equations (S1–S4) online, for the two coordinate systems are obtained by minimising the free energy defined in Eq. (1).

Various techniques can be used to model the non-trivial light propagation for the twist cell in Fig. 1, but Berreman-like methods^27^ are a natural choice for such one-dimensional systems. We use the beam propagation method derived by Oldano^31^ as it allows us to recast the propagation problem as a boundary value problem that can be solved numerically very efficiently using a spectral collocation method^32^. Oldano’s method uses a basis of four waves propagating in a smoothly varying medium: two forward propagating waves with amplitudes $a_1$ and $a_2$, and two backward propagating waves with amplitudes $a_{-1}$ and $a_{-2}$. Maxwell’s equations are solved in matrix form under the following approximations: the refractive index of the medium is a function of only one variable and the incident beam is a plane monochromatic wave. A brief summary of Oldano’s method, as well as the implementation of the model of a twist liquid crystal cell in a solver, is given in the Supplementary information online. The light propagation code is validated by comparison to a COMSOL model that solves Maxwell’s equations for a twist-cell geometry (see Supplementary Fig. S2 online).

## Polarisers orientation

**Figure 2:**
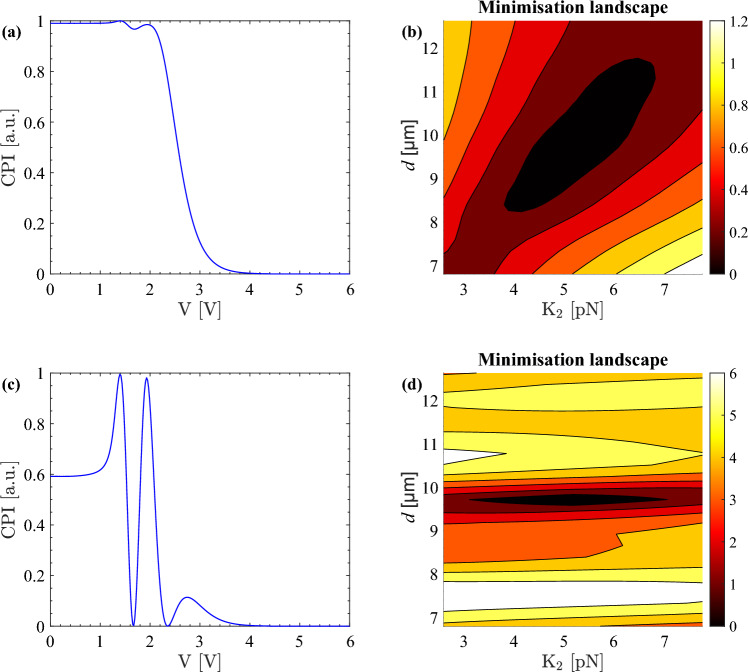
Twist-cell CPI (**a**, **c**) and corresponding minimisation landscape for $\text {K}_2$ and thickness (**b**, **d**) for standard (**a**, **b**) and new (**c**, **d**) experimental configuration. The E7 parameters used for producing this figure are obtained from fitting experimental data taken at $\lambda =642$ nm and have the following values: $\text {K}_1=10.64$ pN, $\text {K}_2=5.18$ pN and $\text {K}_3=16.50$ pN, twist-cell pretilt $\theta _0=1.94^\circ$ and twist-cell thickness $d={9.7}$ μm.

**Figure 3:**
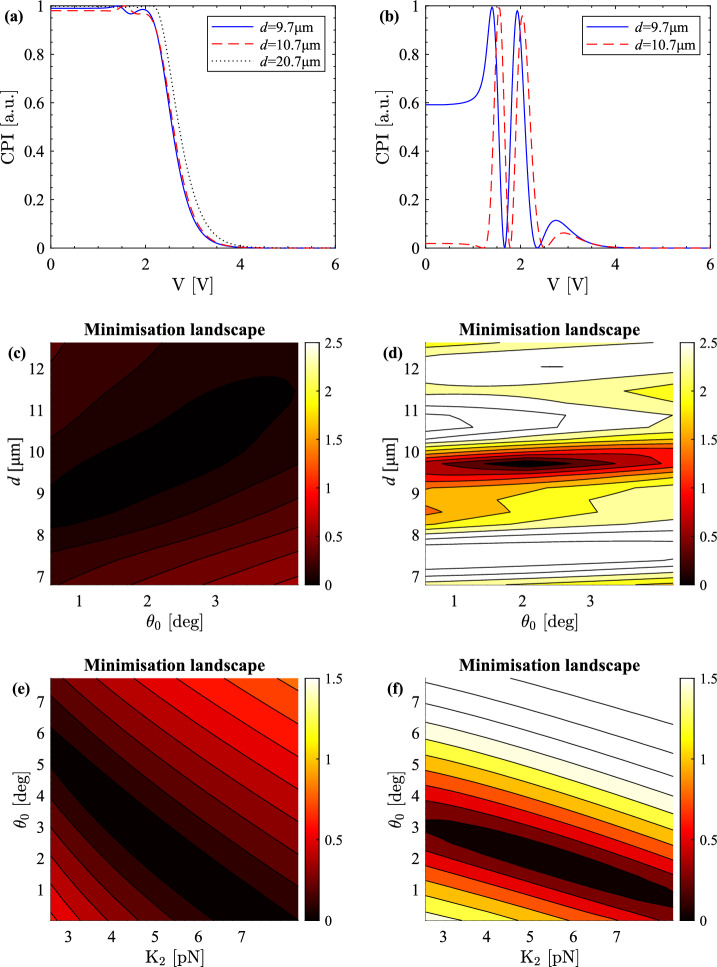
Simulated CPI computed for a selection of cell thicknesses (**a**, **b**), minimisation landscape for pretilt and thickness (**c**, **d**) and minimisation landscape for $\text {K}_2$ and pretilt (**e**, **f**) for crossed polarisers at $0^\circ$ (**a**, **c**, **e**) and $45^\circ$ (**b**, **d**, **f**) to the liquid crystal director. The E7 parameters used for producing these plots are obtained from fitting experimental data taken at $\lambda =642$ nm and have the following values: $\text {K}_1=10.64$ pN, $\text {K}_2=5.18$ pN, $\text {K}_3=16.50$ pN, and twist-cell pretilt $\theta _0=1.94^\circ$. The thickness for the minimisation landscapes is $d={9.7}$ μm.

As already stated, a common approach for the optical analysis of a twist liquid crystal cell is to place the cell between crossed polarisers whose axes are parallel to the liquid crystal molecules at the cell boundaries. This is the initial configuration considered here. The simulated CPI of a twist E7 cell as a function of the amplitude of the applied sinusoidal voltage is shown in Fig. 2a. This is a typical twist-cell CPI trace with well-defined full transmission and no transmission regions. For this configuration of a twist cell and crossed polarisers a variation in the cell thickness does not affect the full transmission and no transmission regions, see Fig. 3a, where the CPI for three different thicknesses is computed. The changes in the CPI trace are moderate even when the cell thickness is doubled. This is ideal for liquid crystal device operation because variation in the thickness of the liquid crystal layer does not hinder the performance of the device.

However, this configuration is not a good choice for the characterisation of twist liquid crystal cells as the CPI trace is fairly featureless. Furthermore, when experimental data are taken, the small features of the CPI can be easily masked by noise. Moreover, in this configuration it is not possible to separate the effect of the twist elastic constant and of the cell thickness on the CPI trace. We show this using a minimisation landscape, i.e. a contour plot of the distance between the original (experimental) CPI data and a theoretical CPI curve computed for a range of values of some of the model’s parameters when all the others are kept fixed. In the case of Fig. 2b, for example, we changed only the twist elastic constant $\text {K}_2$ and the thickness *d* of the CPI trace in Fig. 2a.

The darkest area of the plot in Fig. 2b represents the pairs of $\text {K}_2$ and cell thickness that minimise the distance function and thus become a possible solution when fitting the CPI trace in Fig. 2a. A perfect fit corresponds to a minimum with a value of zero. As the darkest area of the plot covers a big diagonal area, the cell thickness and the twist elastic constant are mutually dependent. This means that one cannot measure $\text {K}_2$ unless the cell thickness is known very precisely. This in itself may not be a significant issue because the cell gap of an empty cell may be known quite precisely. However, the thickness may change after filling the cell with a liquid crystal or may vary laterally. In order to capture this, we must lift this degeneracy, similarly to what is done for planar cells^10^.

The cell thickness and the twist elastic constant are not the only degenerate parameters. The cell thickness and the pretilt, as well as the pretilt and the twist elastic constant, are also degenerate, see Fig. 3c,e. Hence, both the cell thickness and the pretilt need to be known precisely in order to measure $\text {K}_2$. This problem can be significantly reduced by using a different configuration of the two polarisers.

Instead of positioning a twist liquid crystal cell between crossed polarisers whose axes are parallel to the director at the cell boundaries, the twist cell is placed between crossed polarisers at 45$^{\circ }$ to the director at the cell boundaries. This is the same configuration as the one used for planar cells^9–11^. The twist-cell CPI trace for the same parameters used for the standard trace from Fig. 2a is presented in Fig. 2c. Unlike the standard twist-cell CPI from Fig. 3a, a small change of the cell thickness results in a visible change of the new CPI curve, see Fig. 3b. The same cell thicknesses are used for the comparison of the thickness effect in Fig. 3a,b, but the largest thickness is omitted for clarity in Fig. 3b.

The minimisation landscape for $\text {K}_2$ and the thickness corresponding to the new CPI curve from Fig. 2c is plotted in Fig. 2d. Comparing the two minimisation landscapes from Fig. 2b,d shows that the new configuration clearly reduces the degeneracy between $\text {K}_2$ and the thickness. Note that the improvement is so good that the scales on the colourbars are different indicating a significant difference in the steepness of the distance function. The bigger range of the scale in Fig. 2d relates to a higher gradient of the distance function towards the minimum of the minimisation landscape. As the minimum is easier to identify, the values of the fitted parameters are extracted more reliably.

The degeneracy between the pretilt and the thickness is also considerably reduced, see Fig. 3c,d. The minimisation landscape for $\theta _0$ and *d* for crossed polarisers at 0$^\circ$ to the liquid crystal director, i.e. the initial (standard) setup, is presented in Fig. 3c and for crossed polarisers at 45$^\circ$ to the liquid crystal director, i.e. the new (non-standard setup), is in Fig. 3d. The same colourbar scales are used for this comparison. The minimisation landscapes involving the cell thickness in Figs. 2d and 3d show that the thickness can be determined individually, reliably and precisely using the new CPI method, thus offering one of the main advantages of the method.

Figure 3 shows the comparison of the minimisation landscapes for $\text {K}_2$ and the pretilt between the standard setup with crossed polarisers at 0$^\circ$ (Fig. 3e) and the new setup with crossed polarisers at 45$^\circ$ (Fig. 3f) to the liquid crystal director. Even though the degeneracy between $\text {K}_2$ and the pretilt is significantly improved, it is still present in the new configuration of the polarisers, which means that it needs to be considered during the fitting and the measurement of the twist elastic constant. For example, in this case variations of 1.5$^\circ$ in the pretilt correspond to a difference in $\text {K}_2$ of up to 2.5 pN. As the low-voltage part of the CPI trace is affected by the pretilt the most, measurements with good agreement between the experimental data and the fit at that part of the CPI trace are selected as reliable for determining the twist-cell pretilt.

## Results

The new optical method is used to estimate the twist elastic constant $\text {K}_2$ from twist-cell CPI measurements for three well-known liquid crystals at room temperature (about 22 °C). The values of the splay and bend elastic constants $\text {K}_1$ and $\text {K}_3$ are needed in order to apply the method, so planar cells with identical alignment layers are used to extract this information. Both ‘in-house’ and commercial planar and twist cells with cell gap of 10 μm are filled with 5CB, 6CHBT or E7 from Sigma-Aldrich/Merck; no dopants are added. In-house cells are prepared from glass slides coated with indium tin oxide (ITO) electrodes, on top of which polyimide (PI) is spin-coated and rubbed to form the alignment layers. All cells are filled with a liquid crystal in its isotropic state and sealed with epoxy adhesive.

During the CPI measurements, a fibre-coupled diode laser of wavelength 450 nm or 642 nm is used to collect data. The liquid crystal cell is placed between crossed polarisers at 45$^\circ$ to the director at the cell boundaries, see Fig. 1. A fraction of the intensity before the liquid crystal cell and after the second polariser is measured by two photodetectors. The ratio between the transmitted light captured by the photodetectors is recorded as a function of the amplitude of the sinusoidal voltage applied to the cell. The CPI trace is then normalised between 0 and 1, and a best fit is used to extract a range of liquid crystal properties. From the planar-cell measurement we estimate $\text {K}_1$, $\text {K}_3$, planar-cell pretilt and thickness. We have also verified that the polar anchoring can be assumed to be infinitely strong (apart from the commercial planar 5CB cell which will be discussed later) and, hence, that the combination of liquid crystal and alignment layer could be used for twist-cell measurements. We have used this information for the twist cells to estimate $\text {K}_2$ and the twist-cell pretilt and thickness. The planar-cell CPI fit needs the following input parameters: the dielectric coefficients $\varepsilon _\parallel$ and $\varepsilon _\perp$ and the refractive indices $n_e$ and $n_o$. The twist-cell CPI fit requires the same parameters plus the splay and bend elastic constants $\text {K}_1$ and $\text {K}_3$ obtained from the planar-cell CPI fit.

**Figure 4:**
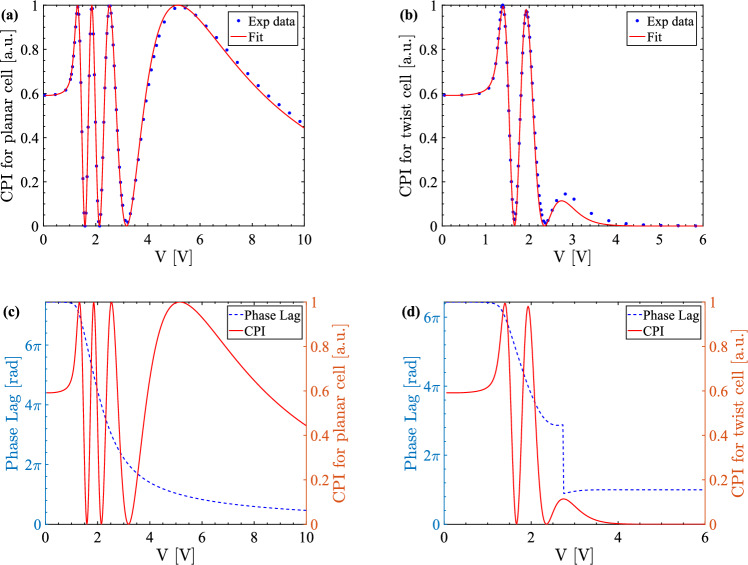
Planar-cell (**a**) and twist-cell (**b**) fit of the normalised CPI for in-house E7 cells with corresponding computed phase lag versus fitted CPI (**c**, **d**). The following values of the elastic constants are measured: $\text {K}_1=10.64$ pN, $\text {K}_2=5.18$ pN and $\text {K}_3=16.50$ pN at $\lambda =642$ nm. The CPI traces given here and the liquid crystal parameters extracted from their fits are used for the analysis in this work.

Figure 4a,b shows typical CPI traces of a pair of planar and twist cells placed between crossed polarisers at 45$^\circ$ to the liquid crystal director, along with their best fits. Both CPI traces are normalised between 0 and 1, and oscillate between these two values with the exception of the last one or two maxima of the twist-cell CPI. The shape of this trace is significantly different from that of a planar cell and indicates that the change in phase lag in planar and twist cells is through different mechanisms; this phenomenon is analysed in the Discussion section. The CPI traces in Fig. 4 belong to in-house E7 cells. The non-optimal fit of the high voltage part of the twist-cell trace in Fig. 4b is a signature that the total twist angle of this cell is not exactly 90$^\circ$. We return to this point later on. Analogous plots of the CPI traces of commercial E7 cells, in-house and commercial 5CB and 6CHBT cells are presented in Supplementary Fig. S3–S7 online. A comparison of the quality of the fits of the experimental CPI traces in commercial and in-house cells, Fig. 4a,b and Supplementary Fig. S3–S7 online, shows that both commercial and in-house cells produce acceptable data with no clear trend of which produce the best fit.

Our results for the splay, twist and bend elastic constants from multiple measurements at room temperature using both commercial and in-house planar and twist cells are summarised in Table 1. The entries in the table show the average value, followed by the range of the measurements in parentheses. The published values of the elastic constants are also included in Table 1. There is a good agreement between the elastic constants measured in this work and the literature values, which indicates the success of the CPI method. The results for $\text {K}_1$ and $\text {K}_3$ for 5CB are slightly outside of the literature values ranges, but that could be due to the low clearing temperature of 5CB (about 35 °C). As the temperature approaches the clearing point and the scalar order parameter reaches zero, the change in the birefringence and the dielectric anisotropy becomes more rapid. This effect, compounded with the temperature dependence of the elastic constants, may result in a bigger variation of the obtained values of the elastic constants as the temperature, at which the measurements are taken, is not precisely known.

**Table 1:** Average and ranges of (in brackets) values measured at room temperature (about 22 °C) and corresponding ranges of literature values of the elastic constants $\text {K}_1$, $\text {K}_2$ and $\text {K}_3$ of three liquid crystals: 5CB, 6CHBT and E7.

LC	$\text {K}_1$ (pN)		$\text {K}_2$ (pN)		$\text {K}_3$ (pN)	
	Measured	Literature	Measured	Literature	Measured	Literature
5CB	5.6 (5.4–5.8)	5.9–6.8^33^	3.7 (3.1–4.1)	3–6^34,35^	8.2 (7.5–8.8)	8.5–10^33^
6CHBT	6.8 (6.8–6.9)	6.2–7.9^33^	3.5 (2.5–4.5)	2.9–3.7^33^	8.5 (8.1–8.9)	7.4–10.6^33^
E7	10.8 (10.5–11.0)	10.5–11.7^36,37^	6.2 (5.2–7.2)	4.1–8.8^12,15^	16.0 (15.5–16.5)	12.3–19.5^12,37^

The spreads of the values for the elastic constants are obtained using multiple measurements of at least one in-house and one commercial cell at two different wavelengths, 450 nm and 642 nm.

It is interesting to note here another phenomenon that could also be caused by the low clearing temperature of 5CB. The polar anchoring for all cells is expected to be strong, but the CPI fits for the commercial planar 5CB cell show a consistent weaker anchoring between $2.0 \times 10^{-4}$ J/m^2^ and $2.5 \times 10^{-4}$ J/m^2^ over multiple measurements. This could possibly be explained by the fact that the polar anchoring energy of 5CB on rubbed PI decreases as the clearing point of 5CB is approached^38^.

## Discussion

Our analysis of the new CPI method for standard twist liquid crystal cells is divided into three parts: first, we investigate the shape of the twist-cell CPI curve, then, we study the dependence of the twist-cell CPI trace on the misalignment between the twist liquid crystal cell and the polarisers, and finally, the effect of a twist angle different from 90$^\circ$.

### Shape of the twist CPI curve

In a planar and a pure twist cell with input polarisation at 45$^\circ$ to the director field, the CPI, *I*, is given by

2 $$\begin{aligned} I = \sin ^{2} \left( \frac{\Delta \Phi }{2} - \Phi _{0} \right) , \end{aligned}$$

where $\Delta \Phi$ is the total phase lag acquired by the field amplitude during propagation across the cell and $\Phi _{0}=\{0,\pi /2\}$ for a planar and twist cell respectively. In a planar cell the phase lag decreases smoothly with the increase of the voltage and, as a consequence, the CPI oscillates between 0 and 1, see Fig. 4c. In a twist cell the phase lag has a more complex behaviour. At low voltage, it decreases smoothly and, as in a planar cell, the CPI oscillates between 0 and 1, see Fig. 4d. However, at large enough voltage, the cell cannot be considered as having a pure twist and Eq. (2) is not valid. The phase lag has a very small increase before decreasing discontinuously by $2 \pi$ and the CPI has a corresponding maximum that is smaller than 1. Both the planar and twist scenarios are compatible with the fact that at very high voltage the phase lag must be such that the CPI is zero, because the liquid crystal is fully aligned with the electric field and the light propagating through the cell sees an isotropic material with refractive index $n_{o}$. However, it is clear that the polarisation dynamics of the twist cell is much richer than that of a planar cell.

**Figure 5:**
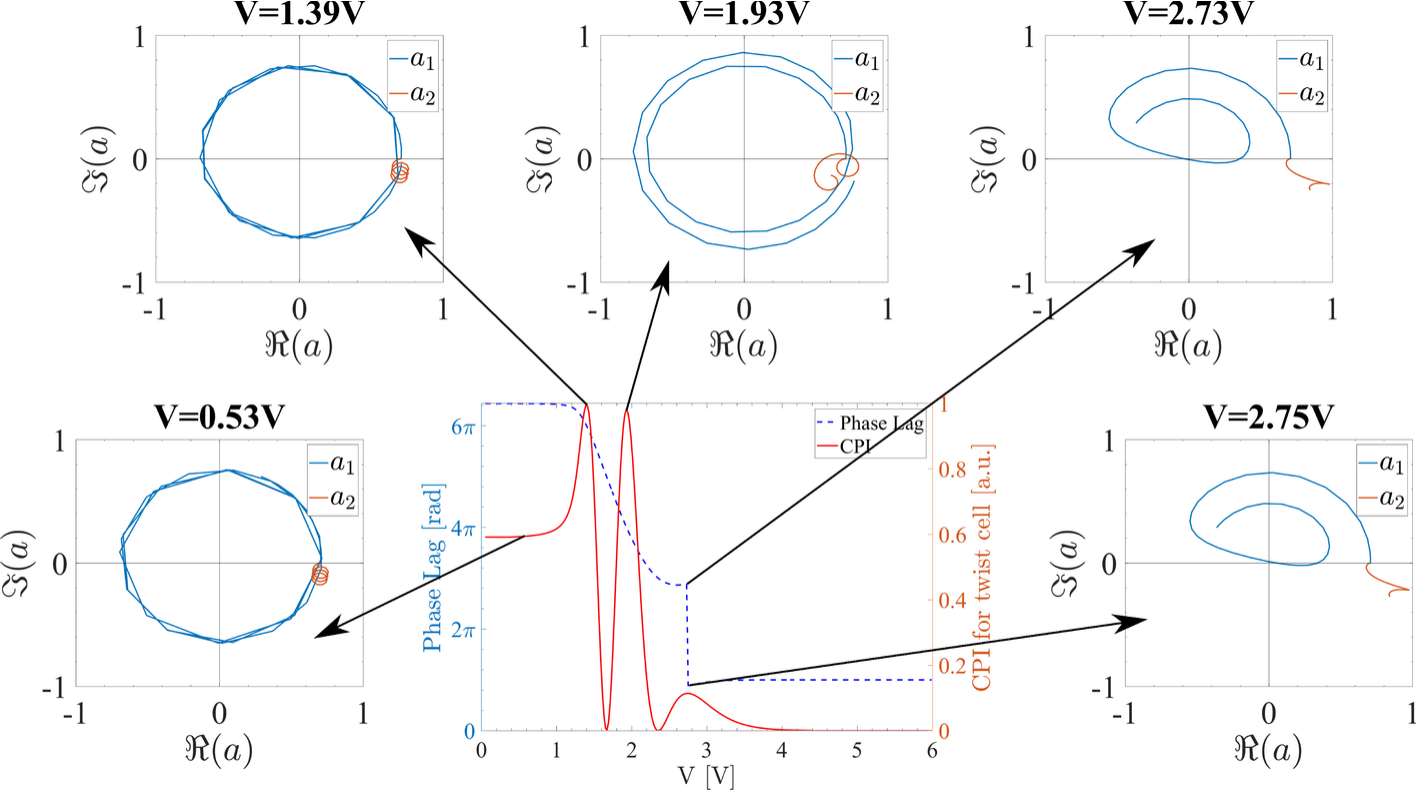
Phase lag components evolution as a function of voltage applied to a twist cell between polarisers at 45$^\circ$ to the liquid crystal director. Here, the Argand diagrams of the amplitudes $a_1$ (blue) and $a_2$ (red) of the forward propagating fields in the cell at five different voltage amplitudes are presented. The amplitudes $a_1$ and $a_2$ are functions of the position in the liquid crystal cell along the *z*-axis which means that they start and end at the liquid crystal cell boundaries.

To understand this difference between planar and twist cells we must analyse the propagation modes of the light through the cell. In a planar cell there are two modes, ${\varvec{e}}_{1}$ and ${\varvec{e}}_{2}$, both fixed in space, one parallel and one perpendicular to the director field plane. Their respective complex amplitudes are

3 $$\begin{aligned} a_{1} = a_{1,0} e^{i k n_{\textrm{eff}}(z) z} \quad \text {and} \quad a_{2} = a_{2,0} e^{i k n_{o} z}, \end{aligned}$$

where $a_{j,0}$ are amplitude coefficients, *k* is the light wavenumber and $n_{\textrm{eff}}(z)$ is the effective refractive index. Its value depends on the orientation of the liquid crystal and decreases with voltage. At low voltage, when the liquid crystal is planar nearly everywhere, $n_{\textrm{eff}}(z) \simeq n_{e}$. At high voltage the liquid crystal in nearly uniformly homeotropic and $n_{\textrm{eff}}(z) \simeq n_{o}$. The total phase lag between the two components is proportional to the difference between $n_{\textrm{eff}}$ and $n_{o}$ and so it is a monotonically decreasing function of voltage, as in Fig. 4c. The amplitude coefficients $a_{j,0}$ are constant and for an input field at 45$^\circ$ to the director field can be normalised to $a_{j,0}=1/\sqrt{2}$. We can represent them pictorially in the complex plane rotated by $\exp (-i k n_{o} z)$. In this rotating frame $a_{2}=1/\sqrt{2}$ is constant while $a_{1}$ draws a circle around the origin with winding number proportional to the total phase lag (the plot is similar to the $V={0.53}\,\text {V}$ in Fig. 5). As the voltage is increased, the total phase lag decreases with the net effect that $a_{1}$ unwinds itself.

This picture is altered only slightly in a pure twist cell, i.e. a twist cell at $V=0$. The only difference is that the modes ${\varvec{e}}_{j}$ are no longer constant but they twist with the director field. As the voltage is increased, the alignment changes from pure twist and this description changes. In particular, it is no longer possible to identify the modes ${\varvec{e}}_{j}$ in terms of the director plane and the amplitudes $a_{j}$ are no longer constant. The effect is small at low voltage, see Fig. 5, where the evolution of the complex amplitudes $a_1$ and $a_2$ is shown at interesting points of the CPI trace and the corresponding phase lag. In the panels $V={0.53}\,\text {V}$ and $V={1.39}\,\text {V}$, $a_{1}$ rotates around the origin and $a_{2}$ is approximately constant. As the voltage is increased the variations in the amplitudes of the two modes become more significant ($V={1.93}\,\text {V}$ panel in Fig. 5): it is no longer true that the energy of the light field is equally divided between the two modes and Eq. (2) becomes less and less valid (note that the CPI maximum at $V={1.93}\,\text {V}$ is slightly smaller than 1). Between $V={2.73}\,\text {V}$ and $V={2.75}\,\text {V}$ all the energy has moved from the first to the second mode, $a_{1}$ goes through zero, the phase lag jumps by $2 \pi$ and the CPI has a maximum that is considerably smaller than 1. This shows why the phase lag dynamics is richer in a twist cell than in a planar cell. In the latter the energy of the two modes is conserved and the only dynamics possible is the unwinding of $a_{1}$. In a twist cell, the energy moves between the modes, $a_{1}$ can cross the origin causing phase lag jumps and a range of maximum values of the CPI are possible.

### Misalignment dependence

**Figure 6:**
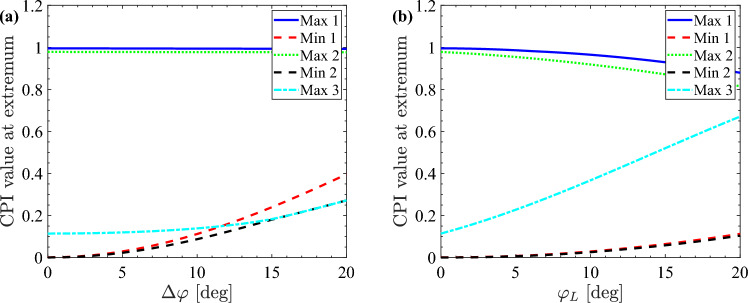
Simulated misalignment angle $\Delta \varphi$ dependence (**a**) and varying twist angle $\varphi = {90}^\circ -\varphi _L$ dependence (**b**) of the value of the CPI at the minima and the maxima of the twist-cell CPI from Fig. 4.

We now consider the effect of a possible misalignment between the twist liquid crystal cell and the crossed polarisers. The CPI measurements are more sensitive to the correct alignment of the cell than in the case of a planar cell. When the orientation of the liquid crystal molecules at the cell surface is not at exactly 45$^\circ$ to the polariser orientation, the minima of the CPI trace do not reach zero, while the tail of the CPI does. This is illustrated in Fig. 6a, where the values of the minima and the maxima of the trace in Fig. 4b are presented as a function of the misalignment angle $\Delta \varphi$, resulting in an angle of ${45}^\circ \pm \Delta \varphi$ between the director at the twist liquid crystal cell boundary and the axes of the crossed polarisers. Figure 6a shows that the CPI maxima do not depend on the misalignment, but the minima do, which produces a detectable variation of the CPI minima for misalignment angles larger than a few degrees. Therefore, when taking experimental data, care must be taken in aligning the liquid crystal cell to ensure that the minima of the CPI trace are as close to 0 as possible.

### Twist angle evaluation

If the twist angle of the cell $\varphi =\varphi _R-\varphi _L$, where $\varphi _L$ is the azimuthal angle at the left liquid crystal boundary and $\varphi _R$ is the azimuthal angle at the right liquid crystal boundary, is not exactly 90$^\circ$, the minima of the CPI trace are only marginally affected. There is, however, a noticeable difference in the height of the last maximum, see Fig. 6b. Here, the dependence of the values of the minima and the maxima of the CPI trace in Fig. 4b on the twist angle of the twist liquid crystal cell is shown. This strong dependence on the twist angle is the reason for the relatively poor fit of the high-voltage part of the twist-cell CPI trace in Fig. 4b. When the twist angle is not exactly 90$^\circ$ and there is misalignment between the liquid crystal cell and the polarisers, it may be hard to distinguish between these two effects on the CPI. However, for small misalignment angles the CPI is relatively insensitive to the alignment of the polarisers, see Fig. 6a, and the only significant effect is observed when the twist angle is not 90$^\circ$, see Fig. 6b.

**Figure 7:**
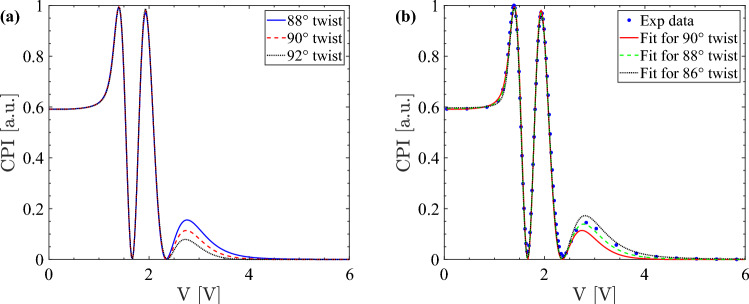
Computed CPI for different twist angles (**a**) and twist angle dependence of the twist-cell CPI fit (**b**). The twist E7 cell parameters and experimental data are taken from Fig. 4b.

Based on this analysis, the measurement method proposed here can also be used for determining the total twist angle of a twist liquid crystal cell, see Fig. 7, where the twist-cell CPI trace from Fig. 4b is further investigated. As it is quite possible that the twist angle of the in-house E7 cell is not 90$^\circ$, the CPI has been computed for a selection of twist angles, see Fig. 7a. Here, all liquid crystal parameters are kept constant apart from the twist angle. The CPI is also fitted using a twist angle $\varphi$ ranging from 86$^\circ$ to 90$^\circ$ in Fig. 7b, where some of the fits have been omitted for clarity. The corresponding values of $\text {K}_2$ and the pretilt obtained from the fits are presented in Supplementary Table S1 online. The values for the thickness are not included as there is almost no change in the thickness (about 0.1$^\circ$) for the different twist angles $\varphi$.

As expected, the main visual difference between the traces in Fig. 7 is in the height of the last CPI maximum. If the twist angle is decreased, the height of the last maximum of the CPI curve increases and the fitted trace shifts closer to the experimental trace in Fig. 7b. In this case the fitted value of $\text {K}_2$ increases (see Supplementary Table S1 online). Instead, if the twist angle is increased, the height of the last maximum of the CPI curve decreases, the CPI trace moves further away from the experimental trace and $\text {K}_2$ decreases. The fit for a twist angle of 88$^\circ$ is closest to the experimental curve at the high-voltage part of the CPI in Fig. 7b indicating that this is the most likely value of the twist angle $\varphi$. If the twist is decreased further, the fit worsens including the low-voltage part. Supplementary Table S1 online shows that the value of $\text {K}_2$ increases by about 0.4 pN for each decrease of the twist angle by 1$^\circ$. The twist angle of the other in-house and commercial cells, whose CPI traces are presented in Supplementary Fig. S3–S7 online, is instead closer to 90$^\circ$. This difference in the value of the twist angle is due to the uncertainties that arise from preparing in-house cells.

## Conclusion

We have developed a new, effective and reliable method for determining the twist elastic constant of nematics in a standard twist cell along with its pretilt, thickness and total twist angle in a single experiment. All four parameters, especially the pretilt and the twist angle, are particularly important in the process of fabrication of twist cells.

The new method is based on fitting CPI measurements and uses a non-standard configuration of crossed polarisers and a twist cell, which allows the accurate determination of the twist elastic constant $\text {K}_2$ by reducing the degeneracy between $\text {K}_2$ and the cell thickness. The model for fitting experimental twist-cell data computes the evolution of the liquid crystal cell alignment as an electric field is applied to the cell using two spherical coordinate systems. An efficient beam propagation method is implemented for computing the non-trivial light propagation in the system, which involves not just standard unwinding, as in planar cells, but also phase jumps. In principle, the fitting procedure of the CPI method requires the splay and bend elastic constants $\text {K}_1$ and $\text {K}_3$ (which are measured using a planar cell), the dielectric coefficients $\varepsilon _\parallel$ and $\varepsilon _\perp$ and the refractive indices $n_e$ and $n_o$, but as previously demonstrated not all of these parameters need to be known precisely^39^. In order to limit the effects of the reliance on the parameters measured using a planar cell and the correlation between the twist elastic constant and the pretilt, we take multiple measurements of both planar and twist cells.

The CPI technique was tested on both ‘in-house’ and commercial liquid crystal cells and was shown to work reliably on both types of cells. The twist elastic constant of 5CB, 6CHBT and E7 was measured with a standard error of 3% for 5CB and E7, and 9% for 6CHBT. The extracted values of the twist elastic constant are within the corresponding range of literature values for each of the liquid crystals, confirming the reliability of our approach and its strength as an aid when fabricating and testing cells. It is possible to further extend the CPI method in order to characterise diverse liquid crystal materials, liquid crystal cells with non-uniform properties, weak anchoring and high pretilt angles as shown for planar cells^11,40,41^.

### Supplementary Information

Supplementary Information.

## Data Availability

Data supporting the results presented in this study are available from 10.5258/SOTON/D3125.
