# Supplementary material for: Optical measurements of the twist constant and angle in nematic liquid crystal cells
Source: Sci Rep. 2024 Jul 31;14:17713. doi: 10.1038/s41598-024-68812-x (PMC11292029; doi:10.1038/s41598-024-68812-x)
Supplement: Supplementary file 1 — Supplementary Information. [file 41598_2024_68812_MOESM1_ESM.pdf]

Optical measurements of the twist constant and angle in nematic liquid crystal cells

Supplementary Information

Alignment equations

We use two spherical coordinate systems to solve the liquid crystal (LC) alignment equations in order to avoid coordinate singularities and preserve the norm of the director. In the case of a planar cell, a single spherical coordinate system can be chosen such that coordinate singularities are avoided. However, this is not possible for a twist cell, so two non-coaxial spherical coordinate systems are needed, a technique called grid patching[1–3].

The choice of Cartesian coordinates is such that the LC cell surface is in the xy-plane, while the voltage is applied along the z-axis, and light propagates through the cell along the z-axis as well. Using this Cartesian coordinate system, the two spherical coordinate systems are defined as specified in Fig. S1. Here, θ_1 and ϕ_1, θ_2 and ϕ_2 are the polar and the azimuthal angles for spherical coordinate system 1 and 2, respectively. In this way, the director can always be defined uniquely in at least one spherical coordinate system. In Cartesian coordinates the arbitrary orientation of the polarisers and the twist cell (from Fig. 1) is defined by the following angles in the xy-plane measured from the x-axis: ζ_L for the first (left) polariser, φ_L for the director at the input (left) boundary, φ_R for the director at the output (right) boundary and ζ_R for the analyser (right polariser). The angles φ_L and φ_R are the left and right ϕ_1 azimuthal angles at the LC boundaries when no voltage is applied. These angles determine not only the angular position, but also the twist of the cell, e.g., if $\varphi_L = 0°$ and $\varphi_R = 90°$, then the cell undergoes a perfect $\varphi = \varphi_R - \varphi_L = 90°$ twist.

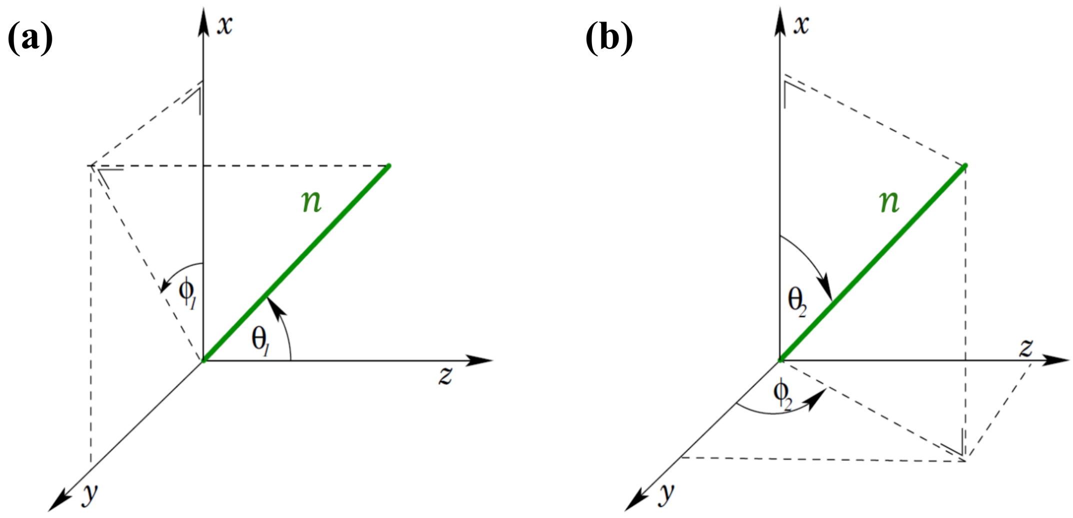

Figure S1. Azimuthal and polar angle definition for the two spherical coordinate systems used for the computation of the director field time derivatives.

The form of the director in the first coordinate system, Fig. S1a, is $\boldsymbol{n} = [\sin\theta_1\cos\phi_1, \sin\theta_1\sin\phi_1, \cos\theta_1]$ and the corresponding non-dimensionalised alignment equations are

$$\begin{aligned}\frac{\partial\theta_1}{\partial t} =& -\frac{1}{2}\delta_3\sin(2\theta_1)\left(\frac{\partial\theta_1}{\partial z}\right)^2 + \left(1+\delta_3\cos^2\theta_1\right)\frac{\partial^2\theta_1}{\partial z^2} \\ & -\frac{1}{2}\sin(2\theta_1)\left[E^2\Delta\varepsilon + \left(1+2\delta_2\sin^2\theta_1+\delta_3\cos(2\theta_1)\right)\left(\frac{\partial\phi_1}{\partial z}\right)^2\right],\end{aligned} \tag{S1}$$

$$\frac{\partial\phi_1}{\partial t}\sin\theta_1 = 2\cos\theta_1\left[1+2\delta_2\sin^2\theta_1+\delta_3\cos(2\theta_1)\right]\frac{\partial\theta_1}{\partial z}\frac{\partial\phi_1}{\partial z} + \left[1+\delta_2\sin^2\theta_1+\delta_3\cos^2\theta_1\right]\sin\theta_1\frac{\partial^2\phi_1}{\partial z^2}, \tag{S2}$$

where $\delta_2 = (\mathrm{K}_2-\mathrm{K}_1)/\mathrm{K}_1$, $\delta_3 = (\mathrm{K}_3-\mathrm{K}_1)/\mathrm{K}_1$ and E is the z-component of the non-dimensionalised applied electric field. The scaling coefficients for the system studied here are the coefficients for the electric field, time and length: V_0, t_0 and L_0, respectively. Their values are as follows:

$$V_0 = \sqrt{\frac{\mathrm{K}_1}{\varepsilon_0}}, \quad t_0 = \frac{\gamma_1 L_0^2}{\mathrm{K}_1}, \quad L_0 = \frac{d}{2}.$$

The scaling factor for voltage V_0 is close to the Fréedericksz threshold voltage, while t_0 is proportional to the LC relaxation time[4]. The last scaling choice L_0 allows the LC boundaries to be selected as $z=-1$ and $z=1$.

In the second coordinate system, Fig. S1b, the director is instead $\boldsymbol{n} = [\cos\theta_2, \sin\theta_2\cos\phi_2, \sin\theta_2\sin\phi_2]$ and the alignment equations become

$$\begin{aligned}\frac{\partial\theta_2}{\partial t} = &\frac{1}{2}\left[\delta_3\sin(2\theta_2)\sin^2\phi_2\left(\frac{\partial\theta_2}{\partial z}\right)^2 + (-2\delta_2+\delta_3-\delta_3\cos(2\theta_2))\sin(2\phi_2)\frac{\partial\theta_2}{\partial z}\frac{\partial\phi_2}{\partial z}\right]\\ &-\frac{1}{4}\left[\Big(2+\delta_3+(2\delta_2-\delta_3)\cos(2\phi_2)+2(\delta_2-\delta_3)\cos(2\theta_2)\sin^2\phi_2\Big)\sin(2\theta_2)\left(\frac{\partial\phi_2}{\partial z}\right)^2\right]\\ &+\frac{1}{4}\left[\Big(2\delta_2-\delta_3+\delta_3\cos(2\theta_2)\Big)\cos(2\phi_2)+\Big(4+2\delta_2+\delta_3-\delta_3\cos(2\theta_2)\Big)\right]\frac{\partial^2\theta_2}{\partial z^2}\\ &+\frac{1}{4}\left[2E^2\Delta\varepsilon\sin^2\phi_2-\delta_2\sin(2\phi_2)\frac{\partial^2\phi_2}{\partial z^2}\right]\sin(2\theta_2),\end{aligned} \tag{S3}$$

$$\begin{aligned}&\frac{\partial\phi_2}{\partial t}\sin^2\theta_2 = \\ &\quad\frac{1}{2}(2\delta_2-\delta_3)\sin^2\theta_2\sin(2\phi_2)\left(\frac{\partial\theta_2}{\partial z}\right)^2+\frac{1}{2}E^2\Delta\varepsilon\sin^2\theta_2\sin(2\phi_2)\\ &\quad+\frac{1}{2}\left[2+\delta_3+2(\delta_2-\delta_3)\cos(2\theta_2)\sin^2\phi_2-\delta_3\cos(2\phi_2)\right]\sin(2\theta_2)\frac{\partial\theta_2}{\partial z}\frac{\partial\phi_2}{\partial z}\\ &\quad+\frac{1}{4}\left[\Big(\delta_2+\delta_3+(\delta_2-\delta_3)\cos(2\theta_2)\Big)\sin\theta_2\left(\frac{\partial\phi_2}{\partial z}\right)^2-2\delta_2\cos\theta_2\frac{\partial^2\theta_2}{\partial z^2}\right]\sin\theta_2\sin(2\phi_2)\\ &\quad+\frac{1}{2}\left[2+\big(\delta_2+\delta_3+(\delta_2-\delta_3)\cos(2\theta_2)\big)\sin^2\phi_2\right]\sin^2\theta_2\frac{\partial^2\phi_2}{\partial z^2}.\end{aligned} \tag{S4}$$

Coordinate system 1 is considered to be the default system as the alignment equations (S3) and (S4) for coordinate system 2 are more complicated than the alignment equations (S1) and (S2) for coordinate system 1. Coordinate system 2 is only used when the director in coordinate system 1 is too close to the coordinate singularity. This transition is specified in the Solver description section.

Oldano's method

Oldano[5] uses Berreman's formalism[6] to describe light propagation in a uniaxial stratified medium by solving Maxwell's equations in matrix form for the case of a plane monochromatic wave with wavenumber k_0 and frequency ω incident in the xz-plane[7]

$$\frac{d\boldsymbol{\psi}}{dz} = ik_0 D\boldsymbol{\psi}, \tag{S5}$$

where $\boldsymbol{\psi}$ is the Berreman vector and D is the Berreman matrix, which are defined as[6]

$$\boldsymbol{\psi} = \begin{pmatrix} E_x \\ H_y \\ E_y \\ -H_x \end{pmatrix}, \tag{S6}$$

$$D = \begin{pmatrix} -\frac{\varepsilon_{zx}}{\varepsilon_{zz}}m & 1-\frac{m^2}{\varepsilon_{zz}} & -\frac{\varepsilon_{zy}}{\varepsilon_{zz}}m & 0 \\ \varepsilon_{xx}-\frac{\varepsilon_{xz}\varepsilon_{zx}}{\varepsilon_{zz}} & -\frac{\varepsilon_{xz}}{\varepsilon_{zz}}m & \varepsilon_{xy}-\frac{\varepsilon_{xz}\varepsilon_{zy}}{\varepsilon_{zz}} & 0 \\ 0 & 0 & 0 & 1 \\ \varepsilon_{yx}-\frac{\varepsilon_{yz}\varepsilon_{zx}}{\varepsilon_{zz}} & -\frac{\varepsilon_{yz}}{\varepsilon_{zz}}m & \varepsilon_{yy}-\frac{\varepsilon_{yz}\varepsilon_{zy}}{\varepsilon_{zz}}-m^2 & 0 \end{pmatrix}. \tag{S7}$$

In these expressions E_x, E_y, H_x and H_y are the x and y components of the complex electric and magnetic field respectively, ε is the permittivity tensor and $m = n_i \sin\theta_i$, where n_i is the refractive index of the incident medium and θ_i is the angle of incidence.

Two different representations are used for the electromagnetic waves: the Berreman vector $\boldsymbol{\psi}$ defined in equation (S6), and another four-dimensional complex vector $\boldsymbol{\phi}$ defined as[7]

$$\boldsymbol{\phi} = \begin{pmatrix} a_1 \\ a_2 \\ a_{-1} \\ a_{-2} \end{pmatrix}, \tag{S8}$$

where a_1 and a_2 are the amplitudes of the two forward propagating waves, while a_{-1} and a_{-2} are the amplitudes of the two backward propagating waves whose polarisation does not change as they propagate through a linear homogeneous medium[5]. These four waves are the eigenvectors of the Berreman matrix D and form a basis. In a continuous stratified medium the relationship between $\boldsymbol{\psi}$ and $\boldsymbol{\phi}$ is given by

$$\boldsymbol{\psi}(z) = T(z)\boldsymbol{\phi}(z), \tag{S9}$$

where T is a 4×4 transformation matrix composed of the eigenvectors of the Berreman matrix D. Using the $\boldsymbol{\phi}$ notation Maxwell's equations from equation (S5) become the propagation equation

$$\frac{d\boldsymbol{\phi}}{dz} = \left(ik_0 D_0 - T^{-1}\frac{\mathrm{d}T}{\mathrm{d}z}\right)\boldsymbol{\phi}, \tag{S10}$$

where D_0 is the diagonal matrix defined by $D = TD_0T^{-1}$. Solving the propagation equation (S10) for $\boldsymbol{\phi}$ in the basis of the propagation modes is simpler than using Maxwell's equations (S5) for $\boldsymbol{\psi}$ as deriving the boundary conditions for $\boldsymbol{\phi}$ is much easier than for $\boldsymbol{\psi}$.

Solver description

In this section the methods and techniques discussed so far are combined into a cross-polarised intensity (CPI) model for a twist LC cell. The transmitted intensity of a twist cell placed between polarisers is computed as a function of the applied sinusoidal voltage. The model was implemented in MATLAB® for the purpose of fitting experimentally measured CPI traces normalised between 0 and 1.

In order to compute the twist-cell CPI the following list of parameters is needed: the extraordinary refractive index n_e and the ordinary refractive index n_o at the laser wavelength λ, the dielectric coefficients $\varepsilon_\parallel$ and $\varepsilon_\perp$, the splay, twist and bend elastic constants K_1, K_2 and K_3, the cell thickness d and the pretilt θ_0. The left and right ϕ_1 azimuthal angles at the LC cell boundaries $z = \pm1$ when no voltage is applied, φ_L and φ_R, are also required, as well as the angles corresponding to the axes of the polarisers, ζ_L and ζ_R. When an experimental CPI trace is fitted, the input (previously known) LC cell parameters are: n_e, n_o,

$\varepsilon_{\parallel}$, $\varepsilon_{\perp}$, K_1, K_3, φ_L and φ_R. The output (fitting) parameters are the twist elastic constant K_2, the pretilt θ_0 and the cell thickness d. During the fitting procedure a series of CPI computations are performed in order to find the best fit of the CPI. This is done by minimising the distance function between the experimentally measured and the computed CPI.

We represent the one-dimensional LC by its director field defined at a number of Gauss-Chebyshev-Lobatto grid points[8] along the z-axis. We use 40 grid points as they are enough to achieve convergence of the calculations performed by our code. The convergence was defined by comparing to calculations over 200 grid points.

We follow the evolution of the LC alignment and the CPI as the applied voltage is increased in steps. In order to do that, we solve the alignment equations in only one coordinate system at each Gauss-Lobatto grid point for each voltage step. We switch between the two spherical systems if we are close to the coordinate singularity in one of the two systems. The threshold to switch between coordinate systems is $\theta_1 < \pi/4$. In this way, we compute the time evolution of the director field, from which we obtain the phase lag using Oldano's method[5] and the CPI. Spatial derivatives are computed using a spectral collocation method[8]. The alignment equations are integrated until equilibrium is reached when the average time variation of the alignment is small enough, i.e. the norm of the time derivative of the polar angle is no longer larger than 10^{-3} rad/s. The code is used in an optimisation algorithm to fit experimental data and estimate the values of the twist elastic constant K_2, the thickness of the cell and the pretilt.

Comparison to COMSOL model

The CPI model for a twist LC cell was compared to a COMSOL model that uses only one of the spherical coordinate systems in Fig. S2. The comparison was made in order to validate the computation of the alignment equations and the approximations of the light propagation model. The comparison between the two models of a twist cell is presented in Fig. S2. The agreement between the CPI computed using COMSOL and the CPI obtained with the new (OMPA) model is good overall but somewhat unsatisfactory at the low voltage part of the CPI. Our analysis shows that this small disagreement is possibly due to ringing fast oscillations of the electric field components in the COMSOL model. These oscillations are probably caused by reflections from the LC layer and the numerical boundaries of the COMSOL model. The OMPA model seems to give more realistic results as it is doubtful that the oscillations present in the COMSOL model exist in a real experimental system.

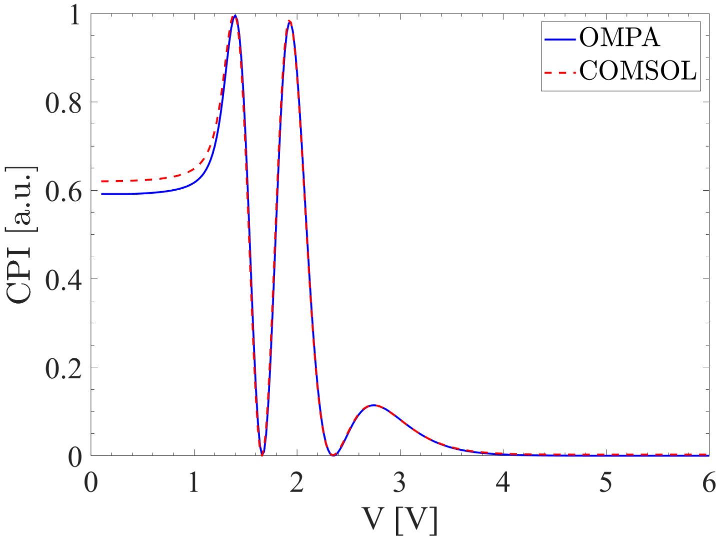

Figure S2. COMSOL model vs OMPA model. The CPI trace is computed using the following E7 parameters: $K_1 = 10.64$ pN, $K_2 = 5.18$ pN, $K_3 = 16.50$ pN, $d = 9.7$ µm, $\theta_0 = 1.94°$, $\varepsilon_{\parallel} = 19.54$, $\varepsilon_{\perp} = 5.17$, $n_e = 1.7287$ and $n_o = 1.5182$ at $\lambda = 642$ nm.

CPI fits of 5CB, 6CHBT and E7

Planar-cell and twist-cell fits of the normalised CPI for in-house 5CB and 6CHBT cells, as well as commercial E7, 5CB and 6CHBT cells, are presented in this section. Figures S3 and S4 show the CPI traces of in-house 5CB cells at 450 nm and 6CHBT cells at 642 nm, respectively. Analogous plots of the CPI traces of commercial E7, 5CB and 6CHBT cells are presented in Fig. S5, Fig. S6 and Fig. S7. The K_2 dependence on different twist angles for the CPI fits of the in-house E7 cell from Fig. 7b is also presented, see Table S1.

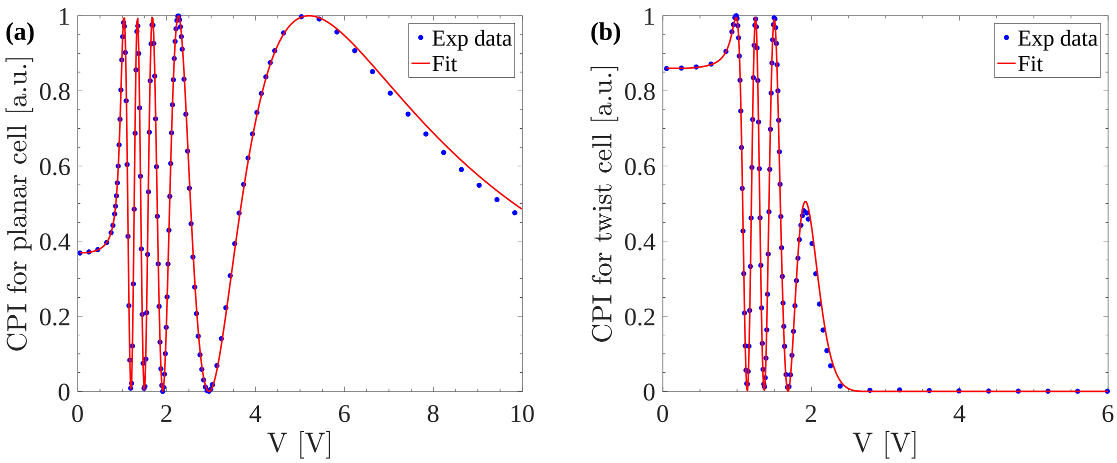

Figure S3. Planar-cell (**a**) and twist-cell (**b**) fit of the normalised CPI for in-house 5CB cells with measured $K_1 = 5.58\,\text{pN}$, $K_2 = 3.62\,\text{pN}$ and $K_3 = 7.83\,\text{pN}$ at $\lambda = 450\,\text{nm}$.

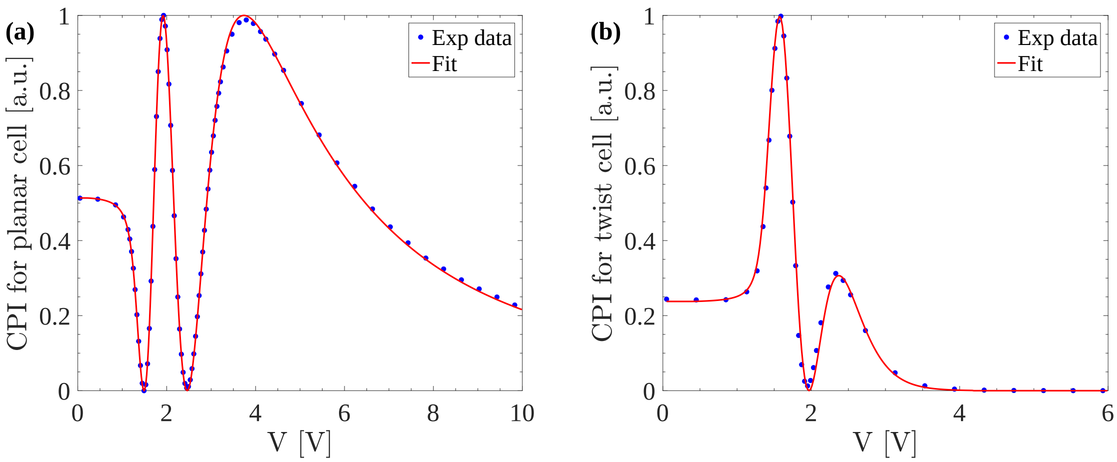

Figure S4. Planar-cell (**a**) and twist-cell (**b**) fit of the normalised CPI for in-house 6CHBT cells with measured $K_1 = 6.83\,\text{pN}$, $K_2 = 4.20\,\text{pN}$ and $K_3 = 8.80\,\text{pN}$ at $\lambda = 642\,\text{nm}$.

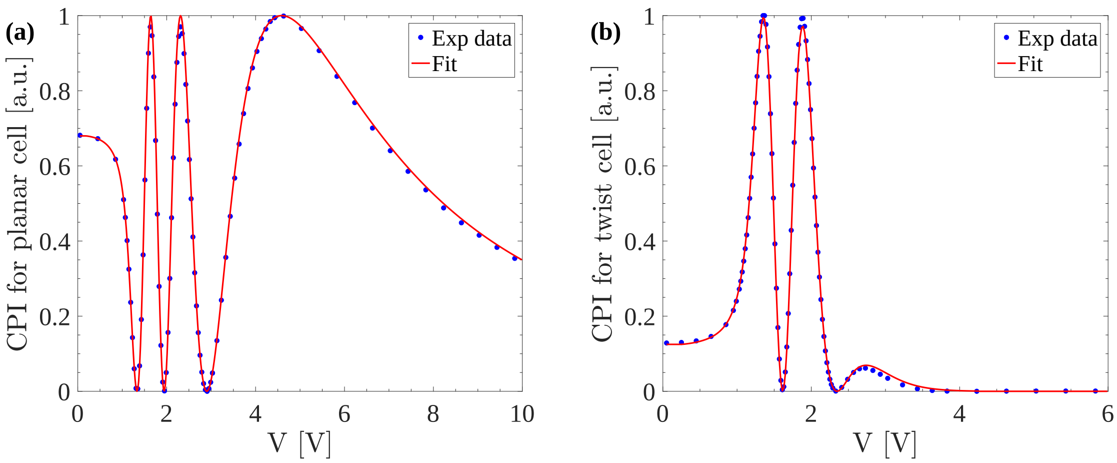

Figure S5. Planar-cell (**a**) and twist-cell (**b**) fit of the normalised CPI for commercial E7 cells with measured $K_1 = 10.91\,\text{pN}$, $K_2 = 6.60\,\text{pN}$ and $K_3 = 15.68\,\text{pN}$ at $\lambda = 642\,\text{nm}$.

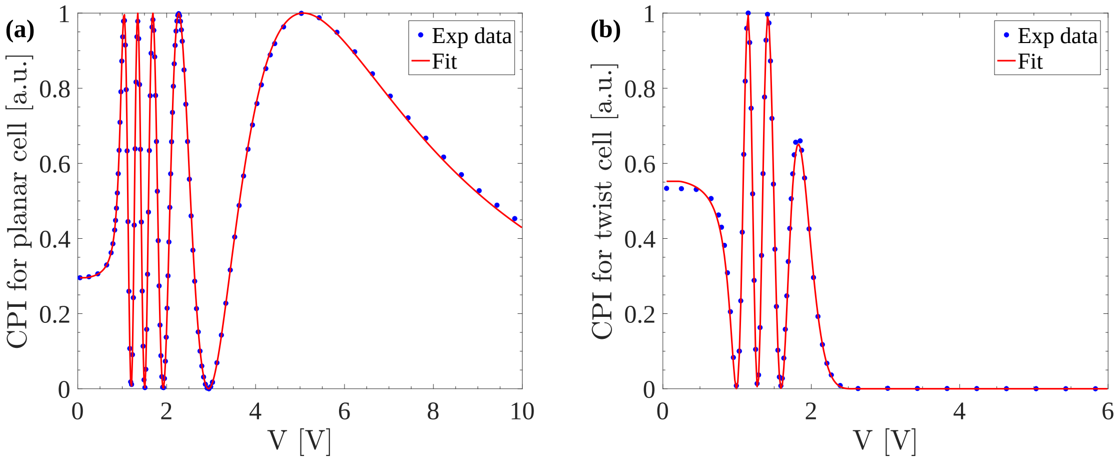

Figure S6. Planar-cell (**a**) and twist-cell (**b**) fit of the normalised CPI for commercial 5CB cells with measured $K_1 = 5.68$ pN, $K_2 = 3.85$ pN and $K_3 = 8.29$ pN at $\lambda = 450$ nm. The polar anchoring of the planar cell is $W_p = 2.5 \times 10^{-4}$ J/m^2. Finite polar anchoring energy of the twist cell is not implemented in the code yet, so strong anchoring is assumed in this case.

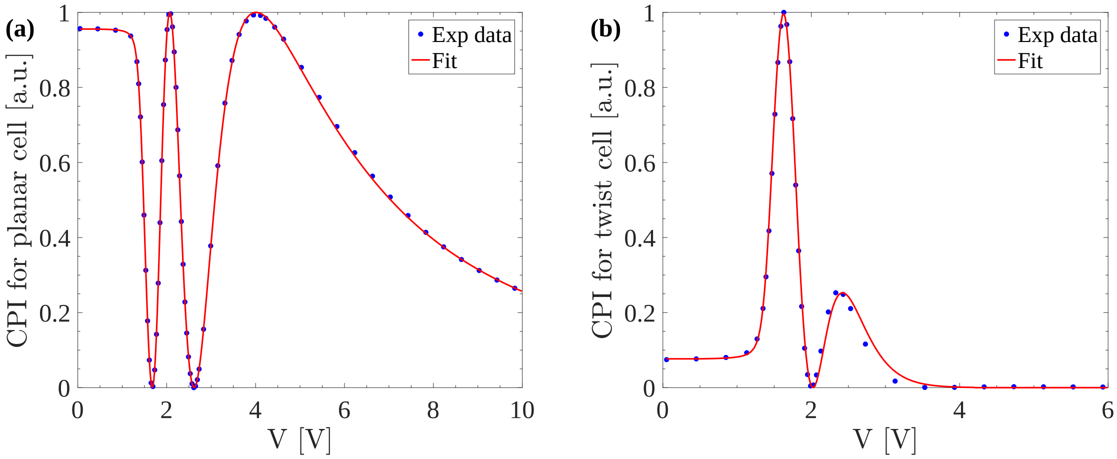

Figure S7. Planar-cell (**a**) and twist-cell (**b**) fit of the normalised CPI for commercial 6CHBT cells with measured $K_1 = 6.90$ pN, $K_2 = 4.48$ pN and $K_3 = 8.68$ pN at $\lambda = 642$ nm.

Dependence of the twist elastic constant on other LC parameters

The CPI method for twist LC cells relies on the values of the K_1 and K_3 elastic constants, so it is necessary to estimate their impact on the measurement of the K_2 elastic constant. In order to address this concern, we computed the minimisation landscape for K_2 and K_3, see Fig. S8. The plot shows that if K_3 is measured with a precision of ± 0.5 pN, the value extracted for K_2 will also differ by up to 0.5 pN from the real value. The minimisation landscape for K_2 and K_1 gives similar estimates. The method also requires the values of the dielectric coefficients $\varepsilon_{\parallel}$ and $\varepsilon_{\perp}$ and the refractive indices n_e and n_o. The accuracy of the CPI method for planar cells depends on the precision of the dielectric coefficients, but not as much on the refractive indices[9]. We expect the same behaviour for the CPI method for twist cells and the twist elastic constant K_2.

φ [°]	K_2 [pN]	θ_0 [°]
86	6.75	1.15
87	6.39	1.32
88	6.02	1.50
89	5.63	1.70
90	5.18	1.94

Table S1. Twist angle dependence of the twist-cell CPI fit of an in-house E7 cell. The values of the twist elastic constant and the pretilt are extracted from the fits in Fig. 7b.

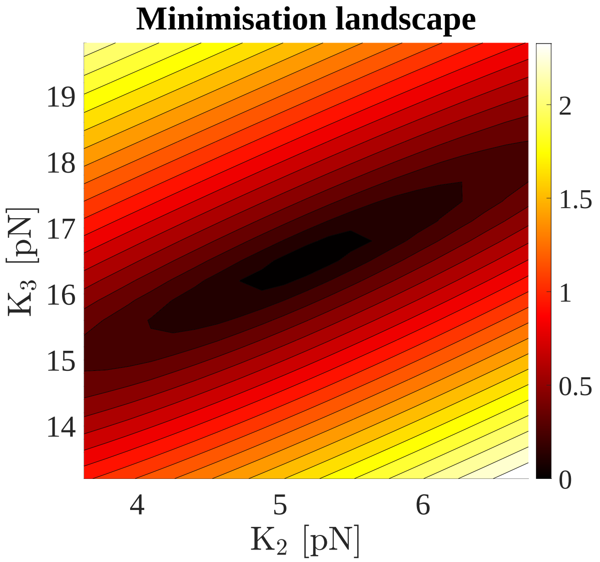

Figure S8. Minimisation landscape for K_2 and K_3. The E7 parameters used for producing this figure have the following values: $K_1 = 10.64\,\text{pN}$, $K_2 = 5.18\,\text{pN}$, $K_3 = 16.50\,\text{pN}$, $d = 9.7\,\mu\text{m}$, $\theta_0 = 1.94°$, $\varepsilon_{\parallel} = 19.54$, $\varepsilon_{\perp} = 5.17$, $n_e = 1.7287$ and $n_o = 1.5182$ at $\lambda = 642\,\text{nm}$.

References

1. Forsey, C. R., Edwards, M. G., Carr, M. P., Edwards, M. G. & Carr, M. P. An investigation into grid patching techniques. NASA. Langley Res. Cent. Numer. Grid Gener. Tech. (1980).
2. Thornburg, J. Black-hole excision with multiple grid patches. Class. Quantum Gravity **21**, 3665–3691, DOI: 10.1088/0264-9381/21/15/004 (2004).
3. Pollney, D., Reisswig, C., Schnetter, E., Dorband, N. & Diener, P. High accuracy binary black hole simulations with an extended wave zone. Phys. Rev. D **83**, 044045, DOI: 10.1103/PhysRevD.83.044045 (2011).
4. Blinov, L. M. & Chigrinov, V. G. Electrooptic Effects in Liquid Crystal Materials. Partially Ordered Systems (Springer New York, 1994).
5. Oldano, C. Electromagnetic-wave propagation in anisotropic stratified media. Phys. Rev. A **40**, 6014–6020, DOI: 10.1103/PhysRevA.40.6014 (1989).
6. Berreman, D. W. Optics in Stratified and Anisotropic Media: 4×4-Matrix Formulation. J. Opt. Soc. Am. **62**, 502, DOI: 10.1364/josa.62.000502 (1972).
7. Elston, S. J. & Sambles, J. R. (eds.) The Optics of Thermotropic Liquid Crystals (Taylor & Francis, 1998).
8. Trefethen, L. N. Spectral Methods in MATLAB (Society for Industrial and Applied Mathematics, 2000).
9. Bankova, D. et al. Characterization of optically thin cells and experimental liquid crystals. Appl. Opt. **61**, 4663–4669, DOI: 10.1364/AO.456659 (2022).
